# COVID-19 pandemic: management of patients affected by SARS-CoV-2 in Rome COVID Hospital 2 Trauma Centre and safety of our surgical team

**DOI:** 10.1007/s00264-020-04715-6

**Published:** 2020-07-15

**Authors:** Domenico De Mauro, Giuseppe Rovere, Alessandro Smimmo, Cesare Meschini, Fabrizio Mocini, Giulio Maccauro, Francesco Falez, Francesco Liuzza, Antonio Ziranu

**Affiliations:** 1 Department of Orthopaedics and Traumatology, Fondazione Policlinico Universitario A. Gemelli IRCCS - Università Cattolica del Sacro Cuore, Largo Agostino Gemelli, 8, Rome, Italy; 2 Santo Spirito in Sassia Hospital, 00193 Rome, Italy

**Keywords:** Pandemic, Trauma, Fractures, COVID-19

## Abstract

**Background:**

SARS-CoV-2 pandemic left a deep mark in the health systems around the globe, leading to an important change in the way we intend the access to the healthcare and its fruition. Hospitals faced something unexpected, and they underwent a deep change and so did orthopaedic activity.

**Materials and methods:**

In “A. Gemelli” University hospital new protocols were adopted for the safe management of patients affected by SARS-CoV-2. Among these patients, six had to be treated also for orthopaedic problems. The management of these patients, from the admission in the Emergency Room (E.R). to the operating room (O.R.), followed the protocols we developed for the coronavirus crisis.

**Results:**

Four among the six patients underwent surgical treatments. Two of them showed a change of their clinical status, due to a worsening of COVID-19 symptoms, so the surgical option was postponed. All of them were admitted to the Infectious Diseases Unit, rather than the Orthopaedic and Traumatology Unit, in order to provide the best measures to prevent the spread of the contagion and to ensure the best treatment for COVID-19. No O.R. staff was infected by SARS-CoV-2.

**Conclusions:**

More studies are needed to provide a higher statistical significance to the safety measures taken in order to contrast the spread of SARS-CoV-2 in the Surgical Room. Orthopaedic surgeons are more exposed to the contagion due to the particular tools set they use. A more sensible and specific quick test for novel Coronavirus is particularly needed, due to the lack of sensitivity of the serological rapid test.

## Introduction

SARS-CoV-2 pandemic left a deep mark in the health systems around the globe, determining an important change in the way we intend the access to the healthcare and its fruition. The disease caused by the novel coronavirus, named COVID-19, determines a higher need for hospitalization in infected patients [1], especially the Intensive Care Unit (ICU) [2].

One of the widest outbreak of the virus was the one happened in Italy, starting from February 20, 2020 [3]. It led the Italian government to impose a nationwide lockdown on March 9 [4]. Italy rapidly became the worst-hit state in the world, overcoming China, the first state to be involved in this epidemic. In Italy there were 231,030 coronavirus cases up to May 27, 2020 [5], and most of them (for some week circa the 40% of the infected) needed a place in a hospital [6], more than 4000 patients were admitted to ICU [7]. In the Lazio region, where our hospital is located, in the same period, there were 7873 diagnosed cases [5].

Italian health system had to develop new protocols for the management of the patients in a short time, to answer the first necessities caused by the situation, for patients’ safety and for health workers’ safety, too. In fact, health workers are the most exposed category in this crisis, with more than 27,000 infected among them in Italy [5]. One of the most prominent changes was with no doubt the management of the patient in the surgical room. There were numerous publications in literature concerning the setting up of new protocols, both for general surgery [8], Trauma Centre, and orthopaedic surgeons [9].

In this article we are going to describe our experience as a Trauma Centre in the management of patients affected by SARS-CoV-2, notably in the peri-operative operations, to allow the right safety to the patient and to the surgical team at the same moment.

## Materials and methods

“A. Gemelli” University Hospital was designed by Lazio region as COVID Hospital: it is the second one in Rome and can be considered among the biggest COVID-centres in the whole country. For surgical urgencies in COVID-19 period, two OR were dedicated to patients, isolated from the rest of the surgical block. Designated guidelines were predisposed for PPEs use in the different places of the hospital, including the surgical block [10].

Our orthopaedic and traumatology unit has seen, for the whole period of the quarantine, a considerable flexability of the admittances in the orthopaedic department, due to the stop of the elective activity, and at the same time, the decrease of E.R. accesses.

From February 21 until today, there were 6 cases of SARS-CoV-2-related patients with a traumatological issue (Table 1). All the patients arrived to our unit from the Emergency Room of our hospital, where they underwent to Rapid IgM/IgG SARS-CoV-2 Test. If the result was positive, the patient was supposed to undergo also to nasal and oro-pharyngeal swabs [11], if not, swabs were only taken if the patient needed to be admitted in the hospital for the prosecution of the healthcare. Swabs were validated according to the actual guidelines [12]. We included only those patients who were considered both affected by COVID-19 or suspected [13], and therefore subjected to the proper procedures for this type of patient.

**Table 1:** Patients affected by SARS-CoV-2 in our Trauma Centre

		COVID-19					
Sex	Age	Anamnesis^1^	Symptoms	Rapid Test	RT-PCR	Diagnosis	Surgery
M	79	Positive	Pneumonia	-	Positive	Aftereffects of left forefoot amputation	Stump revision
M	58	Negative	None	Negative	Positive	Fracture of the left distal radio	Ostheosynthesis with plate and screws
M	64	Negative	None	Negative	Positive	Fracture of II and III finger distal phalanx of the left hand	Ostheosynthesis with K-wires
F	53	Already diagnosed	Fieber > 39 °C	-	Positive	Fracture of the left proximal humerus	Not performed due to worsening of clinical conditions
F	82	Negative	None	Positive	Negative^2^	Left femur pertrochanteric fracture	Intramedullary nail
F	94	Already diagnosed	Fieber > 38 °C	-	Positive	Left femur pertrochanteric fracture	Intramedullary nail

^1^For anamnesis we intended those information about the patient, suggesting a possible SARS-CoV-2 contagion, such as, for example, direct or indirect contact with a known infected subject or an history of travels in red-zones

^2^The patient resulted negative at the first RT-PCR. She was still considered a suspected COVID-19 patient; thus, she underwent to the surgery as an infected patient. She underwent to another RT-PCR test right before the surgery, and the result was again negative. Therefore, she was considered a no-COVID-19 patient and sent to our Unit

Peri-operative safety measures were applied by each member of the surgical team, and all of them underwent to a meticulous dressing and undressing process, as suggested by internal guidelines (Tables 2 and 3), based on the most recent literature about the safety in the orthopaedic activities [14]. All the involved members of the O.R. staff underwent also to the surveillance controls, with the examination of the symptoms and the SARS-CoV-2 test (nasal and oro-pharyngeal swaps).

**Table 2:** Guidelines for the proper dressing before a COVID-patient surgery

Dressing	Switch off the phone and leave all personal belongings in a box outside the operating room
	Wear shoes covers
	Hand hygiene
	Wear surgical cap
	Wear facemask: ○- Surgical mask for surgeons ○- FFP3 respirator mask for the anesthesiologist and the nurses ○- FFP2 respirator mask for instrumentalist of operating room
	Wear goggles or face shield
	Wear lead vest
	Wear isolation disposable gown
	Surgical hand wash
	Wear a first pair of sterile gloves
	Wear a disposable sterile gown
	Wear a second pair of sterile gloves

**Table 3:** Guidelines for the proper undressing after a COVID-patient surgery

Undressing	Remove shoes-covers
	Move with surgical clogs on the green sheet soaked in sodium hypochlorite right ahead the sliding door
	Remove the sterile gown, and simultaneously remove the first pair of sterile gloves
	Remove the disposable gown
	Remove the face shield or the goggles and put it in a container for decontamination
	Remove face mask and surgical cap
	Remove the second pair of gloves
	Leave the operating room
	Wash the hands with hydroalcoholic gel
	It is highly recommended to shower immediately after the procedure and to change the uniform

## Results

All the six patients were sent from E.R. to the Trauma Unit. For each of them there was surgical indication. Two patients came to our hospital already with a COVID-19 diagnosis; thus, they were not submissed to the Rapid Test. Nevertheless, they were tested again, like the others, with nasal and oro-pharyngeal swabs. One of them resulted still positive for SARS-CoV-2, the other, instead, resulted negative, but he was still sent to COVID-Unit. Among the other four patients, three underwent to the Rapid Test first, and then to RT-PCR for SARS-CoV-2. Two of them resulted negative at the Rapid Test, but positive at the RT-PCR. One of them, instead, resulted positive at the IgG/IgM test, but negative at the RT-PCR. For this patient, due to his clinical status and the advices of the anesthesiologist and the infectious disease specialist, he was predisposed an admission to the COVID-Unit anyway. Only one patient came to our Hospital for a pneumonia COVID-19-related, and he was tested only with RT-PCR, with a positive result. All of them were admitted to the Infectious Diseases Unit, waiting for the date of the surgical operation.

Five patients underwent surgical treatments in O.R. for exclusive use of SARS-CoV-2 patients. One patient instead has been suffering of an aggravation of clinical conditions; therefore, the surgical operation were postponed for her. O.R. staff and the surgical team underwent to a dressing- and undressing-process as required from internal guidelines. No intra-operative or peri-operative complications occurred for any of the patients. All the health workers involved in the operations underwent to a control for SARS-CoV-2 contagion through nasal and oro-pharyngeal swaps. No positive results were shown.

## Discussion

Safety protocols for patients with both respiratory and contact isolation are already known by the health workers, especially for those who work in the Operating Room. The wide use of those protocols meets the need of an extraordinary contingency, such as a global pandemic. An invisible enemy, difficult to individuate, makes every health worker afraid of the contagion and for this reason also doubtful on the predisposed safety measures.

Due to the nature of this health crisis, orthopaedic surgeons were not the most involved health workers in this pandemic outbreak; however, they have shown a great capacity of adaptation and solidarity, both helping colleagues in the management of patients in the E.R. [15] and converting their own activity to help the National Healthcare System [16]. The interruption of elective surgery deeply changed the orthopaedic surgeon’s daily routine to allow the hospital to stay focused on those patients, who needed urgent healthcare [17]. As concerns the future of our profession, since we have to deal with the virus, we need to rethink the care pathway within our structures and our services. Traumatological patients affected by SARS-CoV-2 had to be admitted to the Infectious Diseases Unit, while waiting for surgery. This preventive measure was taken for their own safety and for the safety of the other traumatological patients, which were usually elderly and vulnerable.

Coronavirus revolutionized the way we approach clinical cases, pre-operative surgery, and the surgery itself. We have to make our surgical decision not only on the basis of the traumatological evidence but also considering the safety measures to minimize the contagion risk [18].

Orthopaedics and traumatology is a surgical branch that, by definition, has more risk factors for the contagion. Just consider the use of power tools, ultradrive and high-speed burr, determining an increased diffusion of blood in the surgical theatre [17, 19]. The use of Personal Protective Equipment (PPE) and the safety protocols for orthopaedic surgeons have to be a priority.

In our experience, we were able to evidence, even if not with a great experience, a good result according to the safety level reached in our unit. With no COVID-19 cases among the O.R. staff, our team stayed safe even facing directly the contagion in the surgical theatre. Patients affected by SARS-CoV-2 underwent surgery with the same quality and professional standard assured to other non-SARS-CoV-2 patients. Therefore, we reached our primary goal, defending their right to healthcare despite the situation.

In this regard, once the pandemic impact will decrease its force, we need to re-start thinking about patients who are waiting for elective surgery. Due to novel coronavirus, already full waiting lists are going to see even more delays, with an increased discomfort for those patients. A fair balance between emergency surgery and elective surgery at the time of pandemic diseases has to be found.

At last, for a patient with confirmed or suspected SARS-CoV-2, with either clinical, serology, or virological positivity (even if they are not all found at the same time), we need to indicate safety measures in the O.R. since a reliable test with high sensitivity and specificity for SARS-CoV-2 is not available yet.

## Conclusion

The collected data we have just shown here are clearly not enough to have a proper statistical significance, but they allow us to endorse the actual measures for the safety in the hospital setting. None of the O.R. staff members resulted positive at SARS-CoV-2 RT-PCR test after taking part to the surgery. In particular, we need to adopt these protocols in the O.R., to protect the health workers from the contagion, and at the same time, allowing the patients to undergo important treatments they need, without causing any delay because of the coronavirus pandemic. Simultaneously, more studies are needed to validate the use of Rapid IgM/IgG SARS-CoV-2 Test, because at the present time they show a poor correlation with the RT-PCR results.

All patients provided the informed consent for the publication of the clinical history. The study was authorized by the local ethical committee and was performed in accordance with the Ethical standards of the 1964 Declaration of Helsinki as revised in 2000.
